# Short-Term Neural Adaptation to Simultaneous Bifocal Images

**DOI:** 10.1371/journal.pone.0093089

**Published:** 2014-03-24

**Authors:** Aiswaryah Radhakrishnan, Carlos Dorronsoro, Lucie Sawides, Susana Marcos

**Affiliations:** Laboratory of Visual Optics and Biophotonics, Instituto de Óptica “Daza de Valdés”, Consejo Superior de Investigaciones Científicas, Madrid, Spain; Barrow Neurological Institute, United States of America

## Abstract

Simultaneous vision is an increasingly used solution for the correction of presbyopia (the age-related loss of ability to focus near images). Simultaneous Vision corrections, normally delivered in the form of contact or intraocular lenses, project on the patient's retina a focused image for near vision superimposed with a degraded image for far vision, or a focused image for far vision superimposed with the defocused image of the near scene. It is expected that patients with these corrections are able to adapt to the complex Simultaneous Vision retinal images, although the mechanisms or the extent to which this happens is not known. We studied the neural adaptation to simultaneous vision by studying changes in the Natural Perceived Focus and in the Perceptual Score of image quality in subjects after exposure to Simultaneous Vision. We show that Natural Perceived Focus shifts after a brief period of adaptation to a Simultaneous Vision blur, similar to adaptation to Pure Defocus. This shift strongly correlates with the magnitude and proportion of defocus in the adapting image. The magnitude of defocus affects perceived quality of Simultaneous Vision images, with 0.5 D defocus scored lowest and beyond 1.5 D scored “sharp”. Adaptation to Simultaneous Vision shifts the Perceptual Score of these images towards higher rankings. Larger improvements occurred when testing simultaneous images with the same magnitude of defocus as the adapting images, indicating that wearing a particular bifocal correction improves the perception of images provided by that correction.

## Introduction

Presbyopia is the physiological inability to focus near objects that occurs with aging, as the crystalline lens stiffens and loses the ability to reshape upon the accommodative force produced by the ciliary muscle in response to an accommodative stimulus [1]. Multifocal optical corrections such as multifocal contact lenses or intraocular lenses have become an increasingly used solution to restore near vision [2], [3], where certain pupillary regions are corrected for far vision, and other regions have a relative positive power, which allows correction for near. These multifocal solutions produce Simultaneous Vision (SV) wherein a distance correction is superimposed on a near correction creating an overlap of images of the object at the retina at any viewing distance.

Various studies report that the increase in visual performance at near comes at the expense of a degradation of the distance visual performance [3]–[6]. On the other hand, it is traditionally speculated that the optical degradation, produced by the image overlap, is somehow counteracted by the brain and the visual outcome in SV is improved by the suppression of either distance or near image, eventually adapting to the other [7], [8]. However, how the visual system gets adapted to SV has never been tested.

Adaptation and recalibration of the visual system to lower and higher order aberrations have been reported by several studies [9]–[17]. An improvement in visual performance after adaptation to defocus [9], particularly in myopic subjects [10], has also been reported. Also shifts in the isotropic point (the sphero-cylindrical blur producing symmetrical perceived image blur) [11], [12], have been found in subjects after adaptation to images artificially degraded with astigmatism [11], and following astigmatic correction in previously non-corrected astigmats [12]. Studies have also shown that the subjects are adapted to the amount and orientation of blur introduced by the ocular higher order aberrations [13]–[15]. Natural Perceived Focus (NPF) is defined as the amount of image blur producing perception of neither sharpness nor blur (16). Any amount of image blur below the NPF will produce perceptual sharpness and a higher amount of blur will produce perceptual blur. Shifts in the NPF occur after short-term exposure to images blurred with increased or decreased higher order aberrations [16], similar to those demonstrated by Webster et al for artificially blurred or sharpened images [17], [18]. This change in the NPF is considered as a recalibration response of the visual system to any form of blur. Many studies attribute this blur adaptation to a reduction in contrast associated with blur, and therefore, in fact, is a form of contrast adaptation [17]–[20].

Few clinical studies report comparison of visual function on patients implanted with multifocal intraocular lenses or fitted by contact lenses of various designs [21]–[24]. Despite the popularity of multifocal corrections, the impact of simultaneous images on visual performance, and to what extent patients can adapt to simultaneous vision corrections, have been hardly explored. In a recent study, de Gracia et al, using a newly developed Simultaneous Vision Simulator, found that the amount of near addition affected visual acuity differently, with additions around 2 D causing the largest degradation for far vision [25]. However, if and how the brain adapts to the blur pattern produced by simultaneous bifocal vision corrections is still unknown.

The traditional assumption that visual performance with bifocal lenses surpasses the optical degradation imposed by the image superposition, thanks to neural mechanisms that allow suppression of the defocused image [7], [8], is not supported by specific experimental outcomes. We question this interpretation and propose that a deeper understanding of the mechanisms of neural adaptation to multifocality is essential to optimize simultaneous vision designs for the correction of presbyopia. With bifocal corrections, the modulation transfer function decreases non-linearly for higher spatial frequencies, while preserving the contrast at lower spatial frequencies. This difference in the contrast reduction produced by Pure Defocus or Simultaneous Vision was not apparent in the contrast sensitivity measurements in the same subjects measured under either monofocal or bifocal corrections [26].

We hypothesize that the visual system recalibrates to the form and strength of blur imposed by bifocality, following similar mechanisms to those of adaptation to Pure Defocus. In the current study we investigate the extent and amount of neural adaptation to the blur imposed by simultaneous vision, by measuring the visual aftereffects produced following brief exposure to simultaneous bifocal images (with different near additions, and different proportions of far and near vision). The shift in perceived image quality (Natural Perceived Focus and Perceptual Scores) was used as a measure of the neural adaptation and the image quality metrics were used to elucidate the possible mechanisms involved.

## Materials and Methods

### Setup

The experiments were performed using an Adaptive Optics system, which largely compensated the subject's lower and higher order aberrations during the psychophysical measurements. The refractive error of the subject is compensated using a Badal optometer. Subjects' aberrations were measured using a Hartmann-Shack wavefront sensor and were corrected using a membrane magnetic deformable mirror (Imagine Eyes, France). Test and adapting images were presented through a psychophysical channel controlled by the ViSaGe psychophysical platform (Cambridge Research System, UK). The system has a reported correction efficiency of at least 80% in normal eyes. The setup is described in detail elsewhere [27], [28].

### Subjects

The right eye of four subjects, aged 27 to 31 years, with spherical ametropia (<3 D) and astigmatism (<1 D) were measured in the experiment. Overall higher order RMS was 0.79±0.36 *μ*m under natural conditions, and 0.11±0.04 μm after AO-correction. All except one subject were experienced in performing psychophysical experiments.

### Ethics Statement

All protocols met the tenets of the Declaration of Helsinki and were approved by the Consejo Superior de Investigaciones Cientificas (CSIC) Ethics Committee, and subjects provided a written informed consent. The individual photographed has given written informed consent, as outlined in the PLOS consent form, to publication of his photograph.

### Stimuli

Image of a face (480×480 pixels) was blurred by convolution with a Point Spread Function corresponding to different magnitudes of defocus. For Pure Defocus image series (PD), the magnitude of defocus varied from 0 to 2 D in 0.01 D steps.

To generate the simultaneous vision (SV) images, a sharp image (with no defocus) was added to a defocused image. Three different simultaneous vision image series were generated by varying the proportion of the contribution of sharp and defocused images: 25% Sharp and 75% Defocus (25S/75D); 50% Sharp and 50% Defocus (50S/50D), 75% Sharp and 25% Defocus (75S/25D). For example, a 1 D defocus 75S/25D simultaneous image consists of a sharp image (weighted 75%) added to a 1 D defocused image (weighted 25%), and would be equivalent to a bifocal correction of 1 D addition with a 75% of the energy for far, and 25% for near. The magnitude of defocus, in the defocused component in SV images (i.e. equivalent to near additions in a bifocal correction) ranged from 0 to 3 D. All simulations were performed for 5 mm pupil diameter. The images were viewed though Adaptive Optics corrected aberrations and a 5 mm artificial pupil, and subtended 1.98° at the retina, mimicking a subject viewing at far, wearing a full aperture simultaneous bifocal correction, similar to that utilized in diffractive bifocal IOLs. Subjects were presented with an adapting image for 60 s, followed by the test image presentation for 500 ms, after which the subject responded. A re-adaptation was provided between each trial for 3 s.

### Experiments

Simulated images, shown on a CRT monitor, were used to study perceived image quality of and short-term adaptation to simultaneous vision images. To ensure that all subjects had identically blurred images on the retina, the ocular aberrations of the subjects were corrected using the adaptive optics system.

Two experiments were designed to test if the visual system recalibrates after adaptation to SV, like in PD. These experiments evaluated the perception and adaptation to PD and to SV images by measuring the changes in the Natural Perceived Focus and in Perceptual Score. Overall, the experiments lasted for a total of 11 hours and were conducted on two consecutive days with regular breaks in between the sessions.

Natural Perceived Focus (NPF), is the blur that produces a perception of neutrality in blur/sharp vision. A change in the NPF after exposure to a new visual experience (also called aftereffect) accounts for a renormalization of the visual response, so that the adapting stimulus itself appears more neutral, and represents a measure of the short-term adaptation to the new extrinsic context [17], [18]. Perceptual Score defines the perceived image quality of image, and is given by the subject ranging images in a blur-sharp scale.

### Natural Perceived Focus Experiment

**Figure 1:**
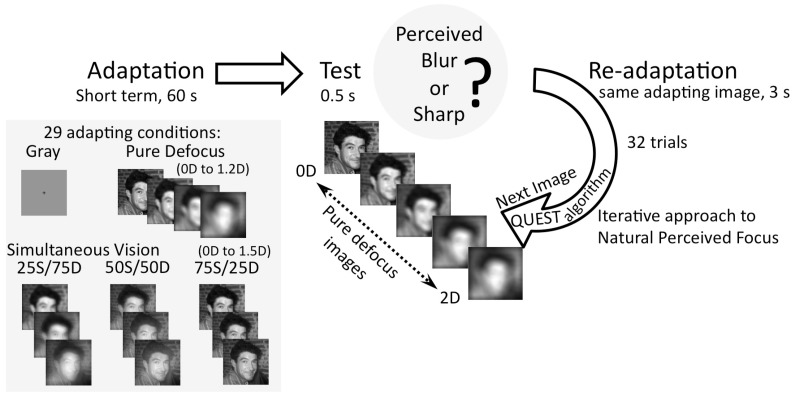
Estimation of Natural Perceived Focus and its change with adaptation. An adapting image is presented for 60(500 ms) for subject's response of blur or sharp (2AFC). The next image for test is chosen based on subject's response using a QUEST algorithm. A re-adaptation image is provided between each test image for 3 s. A total of 32 trials were performed and the average of last 10 stimulus values oscillating around the threshold is defined as the Natural Perceived Focus. Test images were Pure Defocus images. The experiment was done in random sequence for 29 adapting conditions (gray field, sharp, Pure Defocus images of various magnitudes of defocus, and Simultaneous Vision images of different sharp/defocus proportions and magnitudes of defocus).

This experiment was designed to test the effect of adaptation to SV on the NPF. The test images were 201 PD images with defocus ranging from 0 to 2 D, in 0.01 D steps. The adapting images were PD images (6 different levels of defocus between 0.2 and 1.2 D), SV 25S/75D, 50S/50D and 75S/25D images (7 near additions between 0.2 and 1.5 D for each proportion) as well as sharp adaptation condition (defocus = 0) and neutral adaptation with a gray field. In total, 29 adapting conditions were tested. The task for the subject was based on a single stimulus blur detection with a criterion set by the observer [29] coupled with a QUEST (Quick Estimation by Sequential Testing) paradigm of threshold estimation. This adaptive procedure calculates the sequence of stimulus based on initial probability of the threshold and response to actual trial [30]–[31] and was programmed using Psychtoolbox [32]. The subject had to report whether the images presented were blurred or sharp. The QUEST routine usually converged in less than 32 trials, where the threshold criterion was set to 75%. The NPF (expressed in Diopters) was estimated as the average of the 10 last stimulus values, which oscillated around the threshold with standard deviation below 0.01 D (to ensure convergence of the threshold estimate). From previous studies [16], [17], it is expected that the NPF increases when adapting to blur and decreases when adapting to sharper conditions. Figure 1 describes the experimental paradigm and adapting conditions.

The NPF shift was calculated as the difference in the NPF of the adapting image from the NPF after adaptation to a sharp image, equating a 0 D adaptation to a 0 D NPF thereby providing a common reference for all subjects and conditions. The NPF shift was then averaged across subjects. The trapezoidal rule was used to integrate the area under the NPF shift curve up to 1 D defocus in the adapting image for each adaptation condition (PD, 25S/75D, 50S/50D and 75S/25D). The change in area under the NPF shift curve corresponded the overall adaptation and was correlated with the proportion of defocus present in the adapting image series (1 for PD, 0.75 for 25S/75D, 0.5 for 50S/50D and 0.25 for 75S/25D).

### Perceptual Score Experiment

As the optical quality of the images did not vary monotonically with increase in blur in the SV images, a QUEST paradigm, used in NPF experiment, was not suitable to use the SV images as test images. Perceptual scoring experiment allowed testing perception of SV images (50% Sharp image and 50% Defocused image) and how this was altered by adaptation. A control experiment using PD images was performed in order to validate this method as an alternative to estimate NPF.

Series of images with different magnitudes of defocus were presented in a random sequence to the subjects to assess their perceived image quality. The subject's task was to grade the quality of each test image in a 6-point scale, from very blurred (score of 0) to very sharp (score of 5). This procedure was repeated 5 times and the average Perceptual Score was obtained to quantify the perceived image quality for each image.

**Figure 2:**
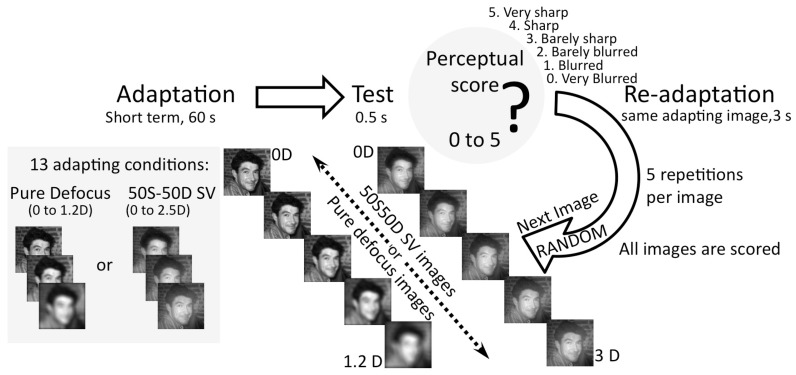
Perceptual Score experiment. Adapting images were presented for 60(500 ms) for subject to score (very sharp to very blurred, in a 6 point scale). Test images were series of 18 Pure Defocus or 19 Simultaneous Vision images presented in a random sequence. The next test image sequence is presented after obtaining subject's Perceptual Score. A re-adaptation image is provided between each test image for 3 s. Subjects adapted to 6 adapting conditions for Pure Defocus (Sharp, 0.25 0.4, 0.6, 1 and 1.2 D) and 7 adapting conditions for simultaneous vision (Sharp, 0.25, 0.5, 1, 1.5, 2 and 2.5 D), in a random order. The Perceptual Score was obtained from the average score of 5 repeated image presentations.

In the control experiment, a series of 18 PD test images (with defocus ranging from 0 to 1.2 D) were presented, and the scoring performed for 6 adapting conditions (sharp image, and 5 PD images with defocus ranging from 0.25 to 1.2 D) in addition to gray adaptation. To evaluate perceived image quality of SV images, subjects scored a series of 19 50S/50D SV test images (with magnitudes of defocus ranging from 0 to 3 D), following adaptation to 7 different conditions (sharp image, and 6 simultaneous vision images with 0.25–2.5 D. Figure 2 describes the experimental paradigm and adapting conditions. Cubic smoothing splines were used to fit the Perceptual Score responses. A smoothing parameter of 0.995 provided a good compromise between oscillation reduction (among contiguous points) and fidelity to the original raw curves. The goodness of the fitting was calculated as the mean difference (in Perceptual Score units) of the experimental data and the spline curves.

For both PD and SV adaptations, the mean Perceptual Score, the Maximum Score Shift and the relative mean Perceptual Score was calculated. For each adapting image, the Mean perceptual score was calculated as the average score for the test images from 0 to 1.2 D. Perceptual Score shift is the difference in Perceptual Score for each adapting condition from sharp adaptation condition. The maximum value of each difference curve (maximum Perceptual Score shift) and the defocus in the test image that produced the largest shift under certain adapting condition were evaluated. Relative mean Perceptual Score was calculated as the ratio of the mean Perceptual Score of the adapting image to the mean Perceptual Score of the sharp image.

#### Image quality metrics

To understand what property of the image drives the perception and adaptation, image quality metrics were calculated. The RMS contrast for each test and adapting images used in Natural Perceived Focus and Perceptual Score experiments were calculated as the standard deviation of the ratio of total luminance and mean luminance in the image, a method previously described by Peli [33]. Computations were performed using custom routines programmed in Matlab (Mathworks Inc). Also, for the same images the Multi-Scale Structural Similarity Index-MSSSIM was calculated. This image quality metric described by Wang et al [34] considers changes in the structural, luminance and contrast components, for multiple scales. In the current study, the sharp image was considered as the reference image and the similarity of the defocused image is calculated from this reference. A Gaussian window of 11 with a standard deviation of 0.5 was used to mimic our experimental perceptual responses. Since the images were the same and differed only in the amount of blur, it can be assumed that the MSSSIM is indirectly related to the contrast degradation, at different scales. Higher values of MSSSIM indicate greater degradation of images. The quality metrics were obtained using ImageJ software [34].

## Results

### Natural Perceived Focus and Its Shift with Adaptation

Natural perceived focus was tested by a single stimulus detection task by using Pure Defocus test images after adaptation to a neutral gray field, sharp image (0 D defocus), and after adaptation to PD and Simultaneous Vision (SV) images.

**Figure 3:**
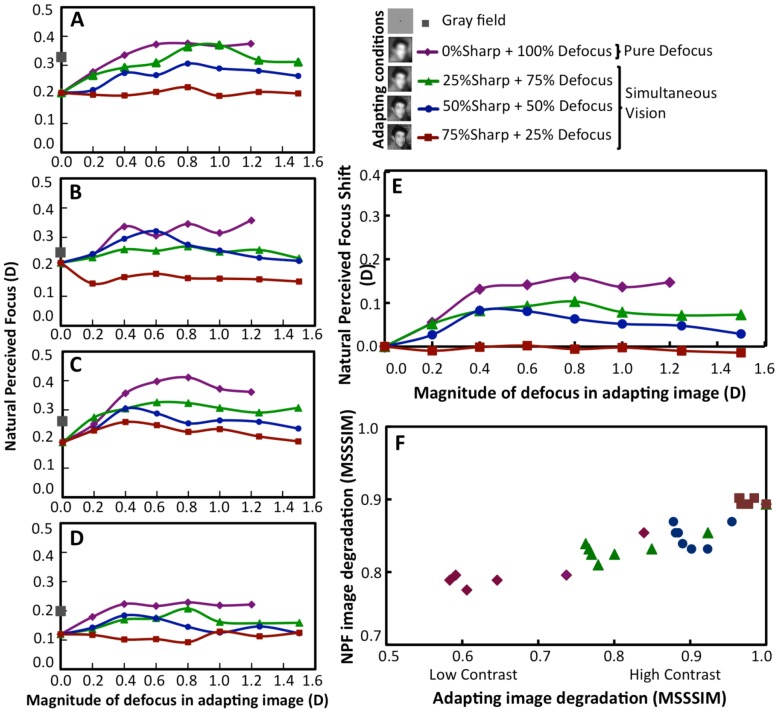
Shift in Natural Perceived Focus after adaptation to Pure Defocus and Simultaneous Vision images. The test images were series of Pure Defocus images. The adapting images were a gray field, Pure Defocus images, and Simultaneous Vision images with various proportions (25%, 50% or 75%) of sharp and blur (25S/75D, 50S/50D, 75S/25D). (A)–(D) show NPF for individual subjects for the different adapting conditions. (E) NPF shifts (differences with respect to the NPF after adaptation to a sharp image) averaged across subjects. Error bars stand for standard deviations. (F) The change in NPF (MSSSIM) with change in MSSSIM of adapting images. Examples of adapting images are given along with the legend.

The NPF, measured using PD test images, varied after adaptation to PD and SV images in all subjects. Figure 3 (A–D, for each of the four subjects) shows the NPF as a function of the magnitude of defocus (expressed in diopters, D) in the adapting image. For PD images this corresponds to the amount of defocus, for SV it is equivalent to the power of addition for near vision in a bifocal correction in SV images. Adaptation to PD images produced the highest shift in the NPF. Adaptation to SV images also produced shifts in the NPF, which varied with the magnitude of defocus and with the proportion of defocus in the adapting image. For example, adapting to 75S/25D simultaneous images (i.e. a combination of 75% sharp image and 25% defocused image) produced little shift of the NPF, whereas adapting to 25S/75D images (25% sharp image and 75% defocused image) produced a shift approaching to that produced by PD images (0% sharp and 100% defocused image). Results are highly consistent across subjects, with slight variations in the magnitude of NPF shifts.

The NPF after adaptation to a gray field varied across subjects (shown as gray squares in Figure 3). In addition, the NPF was not equal to zero after adaptation to a sharp (0 D, fully corrected) image, although it was generally lower than the NPF after gray field adaptation.

Figure 3E shows the NPF shifts (difference in the NPF after blur image adaptation and the NPF after adaptation to a sharp image, expressed in diopters in PD images), averaged across subjects. The sharp image was used as a common reference to all subjects. For PD adapting images, maximum NPF shift of 0.18 D was obtained with a 0.4 D adapting image, and then it saturated. For SV adapting images the maximum NPF shift was 0.08 D and 0.12 D for a 0.4 D 50S/50D and 0.8 D 25S/75D adapting images respectively, and then decreased significantly (p<0.01) for higher defocus values. The area under each average NPF shift curve was used to evaluate the amount of neural adaptation for each adapting condition, the larger the area, the greater the effect of adaptation. There was a highly significant correlation (r = 0.99, p<0.001) between the area under the NPF shift curve and the proportion of defocus component in the adapting images (e.g.: 1 for PD and 0.50 for 50S/50D).

### Natural Perceived Focus Shift and Image Quality

The overall image degradation of the adapting image was described in terms of image quality metrics (RMS contrast and Multi-Scale Structural Similarity index-MSSSIM). The NPF shift with adaptation correlated significantly with the overall image degradation of the PD adapting image. The coefficients of correlation between NPF shift and RMS contrast and NPF shift and MSSSIM for PD adapting images were r = −0.89 (p<0.0001) and r = −0.96 (p<0.0001) respectively. The coefficients of correlation between NPF shift and RMS contrast for 25S/75D, 50S/50D and 75S/25D adapting images were r = −0.80 (p<0.0001), r = −0.23 (p = 0.12) and r = 0.53 (p = 0.002) respectively. Likewise, the correlation coefficients between NPF shift and MSSSIM of adapting images were r = −0.89 (p<0.0001), r = −0.57 (p = 0.0007) and r = 0.41 (p = 0.02) for 25S/75D, 50S/50D and 75S/25D adapting images respectively. Further analysis (Figure 3F) revealed that the process of SV adaptation is partly similar to adaptation to PD. The figure shows that the degradation (MSSSIM) of the image chosen as NPF was highly and significantly correlated (r = 0.95, p<0.0001) with the image degradation (MSSSIM) of the adapting images regardless whether those were PD or SV images. A 50% decrease in MSSSIM of PD adapting images produced an increase in NPF of 150%, or equivalently, a reduction of image quality by half resulted in an increase of NPF by 0.15 D for PD adapting images. Likewise, a reduction in MSSSIM from 1 to 0.9 in SV images resulted in a maximum increase in NPF by 0.1 D.

### Perceptual Score and Its Shift with Adaptation

In this experiment (Figure 2), subjects scored PD and SV test images from very sharp to very blurred, after adapting to gray, sharp, six PD images and seven SV images. A shift in Perceptual Score following exposure to images with different blur is indicative of adaptation, as the same set of images are judged differently depending on the image that the subject has been adapted to.

**Figure 4:**
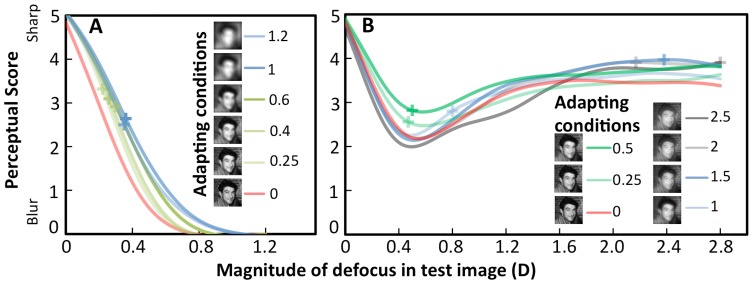
Perceptual Score of Pure Defocus and Simultaneous Vision 50S/50D image. Cubic smoothing splines fit of the Perceptual Score responses of (A) Pure Defocus images as a function of defocus in the test image, after adaptation to Pure Defocus images (with defocus ranging from 0 to 1.25 D). (B) Simultaneous Vision images (50% Sharp and 50% Defocus) as a function of the magnitude of defocus (Near addition) in the test image, after adaptation to simultaneous images with different additions (0 to 2.5 D). The crosses on each curve indicate the images producing maximum after effects. It can be noted that 0.5 D adapting image (red line) produces maximum blur adaptation.

Figure 4 shows the Perceptual Score of the images (cubic splines to the experimental data) as a function of the magnitude of defocus in the test images for PD images (A) or in the defocused component of SV images (B). Data are averaged across subjects for each adapting condition. The mean deviation in Perceptual Score between experimental measurements and fitted curves was 0.017. This deviation is much smaller than the intra/inter-subject variability (SD of 0.4 in the score). The superimposed crosses indicate the defocus values for which each curve deviates most from the sharp adaptation condition (red curve), i.e. the defocus for which neural adaptation produces maximum aftereffects. For PD, those values are around 0.25 D of defocus in PD (Figure 4A). However, for SV, they are scattered across the different defocus component values (Figure 4B). For PD images, increasing the magnitude of defocus in the test image progressively decreased the Perceptual Score. As shown in Figure 3E, there was a very consistent shift of the curves towards higher scores following adaptation indicating that brief exposures to defocused images increase the perceived quality of defocused images. For example, the same 0.4 D defocused image was scored on average close to 1 (blurred) after adaptation to a 0.25 D defocused stimulus, and close to 2.5 (less blurred), after adaptation to a 1 D defocused stimulus.

Unlike with PD images, scoring of the SV 50S/50D test images (Figure 4B) did not decrease progressively with the magnitude of defocus (near addition). While there was a progressive decrease in perceived quality for simultaneous images when near addition increased from 0 to 0.4–0.5 D, the perceived quality increased for higher amounts of addition in the image. Beyond 1.5 D of addition the images were scored above 3, i.e. in the sharp region. Lower additions in the simultaneous vision corrections tend to introduce small phase shifts in the blurred image which further degrade the perceptual image quality. As the blur amount increases, the blurred component of the image tends to become gray (lower spatial frequency content and lower contrast) and the uniform grayness tends to diminish the impact on the perceptual image degradation.

### Effect of Adaptation on Mean Perceptual Score

**Figure 5:**
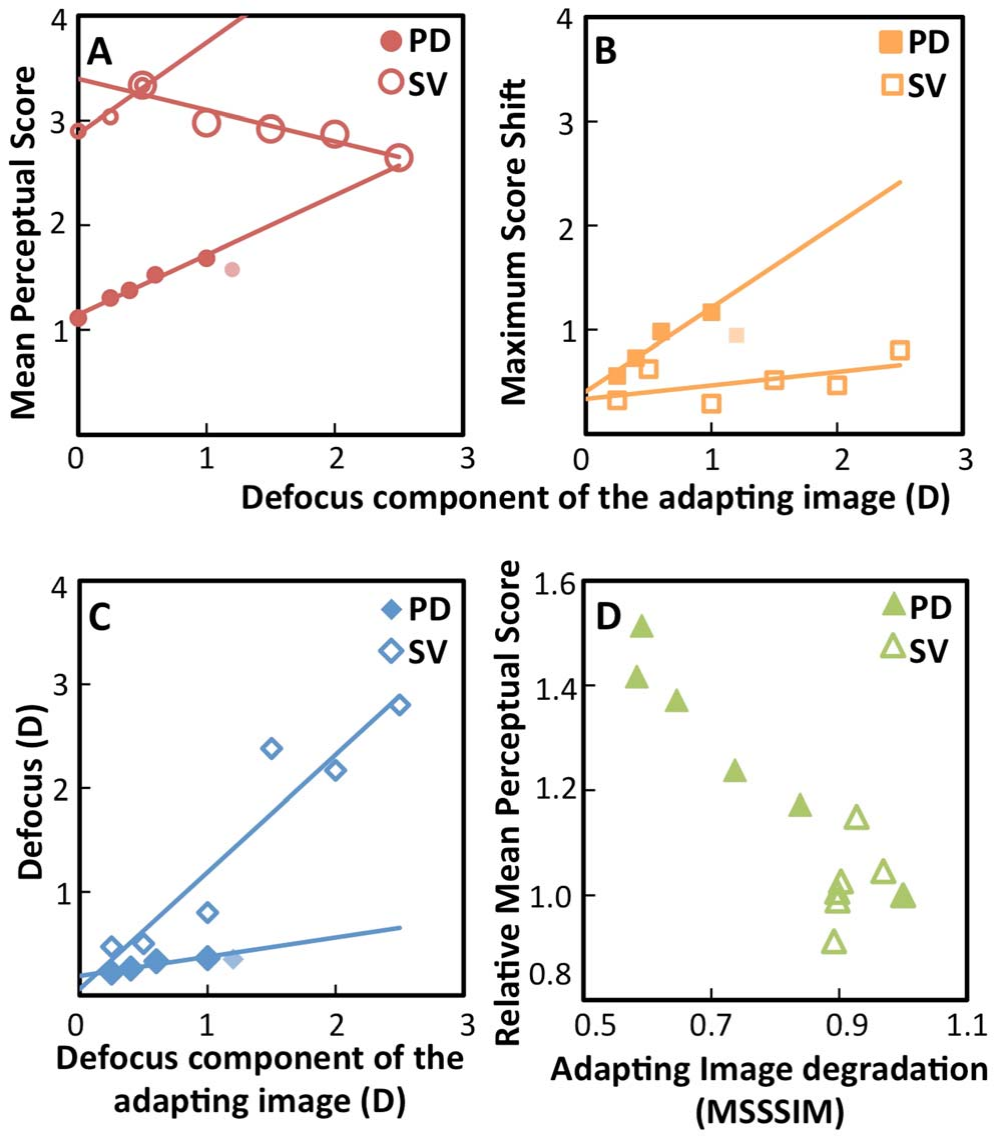
Effect of adaptation on the Perceptual Score. Data are presented as a function of defocus in the adapting image: Defocus for Pure Defocus (obtained from Figure 4A, solid symbols), and magnitude of defocus in the defocused component (near addition) for Simultaneous Vision (obtained from Figure 4B, open symbols). Lines represent linear regressions to the data. Small pale symbols are data after saturation and are not included in the fits. (A) Mean Perceptual Score for test images from 0–1.2 D, as a function of magnitude of defocus in the adapting images. Pure Defocus shows a linear increase (slope 0.57, r = 0.99, p<0.001). Simultaneous Vision shows an initial linear increase (small red open circles, slope = 0.87, r = 0.97, p = 0.13), similar to the Pure Defocus behavior, a maximum at 0.5 D (double circle) and a decrease (large red open circles, slope = −0.3, r = 0.94, p<0.02) for defocus higher than 0.5 D. (B) Maximum shifts in the Perceptual Score (from the Perceptual Score following adaptation to sharp) as a function of defocus in the adapting image, for Pure Defocus (slope = 0.80, r = 0.97, p<0.03) and for Simultaneous Vision (slope = 0.13, r = 0.59, p = 0.2). (C) Defocus in the images that suffered the largest shift in Perceptual Score, as a function of defocus in the adapting image, for Pure Defocus (slope = 0.19, r = 0.945, p<0.06) and for Simultaneous Vision (slope = 1.13, r = 0.94, p<0.005). (D) Relative mean Perceptual Score (mean Perceptual Score of adapting image/mean Perceptual Score of sharp image) as a function of MSSSIM of Pure Defocus and Simultaneous Vision adapting images. There was an initial increase in relative mean Perceptual Score with decrease in the MSSSIM of adapting images.

The mean Perceptual Score was obtained for each adapting condition, by averaging the Perceptual Score of all test images with defocus up to 1.2 D. As shown in Figure 5A, for PD (red solid circles), the mean Perceptual Score increased significantly and linearly with defocus in the adapting image (slope 0.57, r = 0.99, p = 0.001) until it reaches saturation at 1.2 D. The mean Perceptual Scores for SV images were higher than those for PD, but showed a similar trend. An initial linear increase occurred for lower amounts of defocus (small red open circles, slope = 0.87, r = 0.97, p = 0.13), followed by a decrease for higher amounts of defocus (large red solid circles, slope = −0.3, r = 0.94, p = 0.02). These results are in good agreement with the NPF shift results, which showed that, as the adapting defocus increased, the test image with higher blur appeared more focused.

### Effect of Adaptation on the Maximum Score Shift

Figure 5B shows the maximum difference in Perceptual score for each adapting image from the sharp adaptation (Maximum Score Shift), which increase linearly with defocus in the adapting image (slope = 0.80, r = 0.97, p = 0.03 for PD; slope = 0.13, r = 0.59, p = 0.2 for SV). Maximum score shifts were all positive, indicating a recalibration, as blurred images are perceived as sharper after adaptation. If the defocused component of the SV images were suppressed, the Maximum Score Shift would have been all negative, indicative of a sharp adaptation.

### Defocus Values Producing the Maximum Score Shift

Figure 5C represents the defocus values in the test image that produce the maximum shifts in the Perceptual Score under a certain level of adaptation. For both PD (blue solid diamonds) and SV (blue open diamonds) the defocus of test image producing Maximum Score Shift increases linearly with the defocus in the adapting image (slope = 0.19, r = 0.945, p = 0.06 for PD; slope = 1.13, r = 0.94, p<0.005 for SV), indicating high adaptation to the addition required to specific working distance.

#### Perceptual Score and Image quality

The Perceptual Score of the images correlated strongly with image quality degradation when judging PD test images, for both the image quality metrics evaluated (RMS contrast: r = 0.94, p<0.001; MSSSIM: r = 0.99, p<0.0001). However, the Perceptual Scores for SV 50S/50D test images correlated significantly only with the MSSSIM (r = 0.67, p = 0.001) but not with RMS contrast (r = 0.21; p = 0.28), suggesting that local changes in contrast are better predictors of SV perception and adaptation than global contrast. Figure 5D shows the Mean Perceptual Score of the adapting image (relative to the Mean Perceptual Score of the sharp image) as a function of the MSSSIM of adapting images. For PD adapting images (solid green triangles), the relative mean Perceptual Score increased with a decrease in the image quality of adapting image (r = −0.97, p<0.0001). For SV adapting images (open green triangles), the relative mean Perceptual Score increased up to a point corresponding to the highest image degradations (r = −0.99, p<0.0001) and decreased for lower values of MSSSIM (r = 0.92, p<0.0001).

## Discussion

Multifocal optical corrections are becoming popular solutions for compensation of presbyopia, aiming at providing the patient with a range of focus for functional vision at near without compromising far vision [2]–[6]. These multifocal corrections provide a simultaneous image on the retina, ideally one in focus and the other defocused. One of the hypotheses for adapting to simultaneous images implies that the brain suppresses the blurred component of the image, making the image look sharper to the subject than the actual physical degradation produced by superimposition of the images [7], [8]. However, whether this really happens had never been tested.

### Inter-subject Differences in Perception

In our study, the aberrations of the subjects were corrected to a large extent (86% on average) with adaptive optics and the subjects viewed the adapting and test images under similar viewing conditions. NPF after adaptation to a gray field differed across subjects, as previously reported by Sawides et al [13], [16]. The measurement of the NPF under neutral adaptation (gray field) has been shown to match the NPF under natural viewing conditions (adapting image only degraded by the natural aberrations of the subjects). This stimulus level that corresponds to the perceptual norm of the subject (internal code of blur) varies across individuals, driven by the amount of blur produced by the aberrations of their ocular optics [13]. Therefore, the differences in natural perception (pre-adaptation states) across the subjects of the study are most likely associated to the differences in their ocular optics (and therefore in the internal code for blur). However, these individual differences were substantially reduced when subjects were instead adapted to a common stimulus in the experiment, with the shifts in the NPF and in the Perceptual Scores of the subjects following a similar trend upon adaptation (Figure 3A–D), which indicates that the recalibration of the internal code for blur follows similar patterns across individuals.

### Simultaneous Vision vs Pure Defocus

Adaptation to Simultaneous Vision (SV) images produced a shift in the NPF similar to that produced by purely defocused images, although of lower magnitude. Simultaneous images are objectively less degraded than pure defocus images. Charman et al. [26] showed that the high spatial frequency content is retained in a bifocal blur, and therefore simultaneous vision images appear optically less degraded than pure defocus images. We found that the NPF shift was mostly influenced by the proportion and magnitude of the defocus present in the adapting image. For instance, adapting to a simultaneous image with 75% of defocus (and only 25% of sharp image content) produced somewhat similar aftereffects to those produced by Pure Defocus (PD).

The NPF and mean Perceptual score results were concurrent. There was a linear relation between the NPF shift (and Perceptual Score shift) with the magnitude of defocus in the adapting images, following adaptation to PD images. This effect of adaptation to PD was consistent across the two experiments (Figure 3E, 4A), as well as with previous studies [9], [13], [17]. The maximum NPF shift when adapting to SV images occurred for a magnitude of defocus in the defocus component of around 0.5 D, which was also, interestingly the SV image that was scored as more blurred in the Perceptual Score experiment, despite the test images being different in the experiments. The higher slope of the PD curve compared to the SV curve in the maximum score shift is indicative of the higher adapting effect of PD images.

### Theories of Adaptation to Simultaneous Vision

Traditionally, adaptation to SV images has been interpreted as a suppression of the defocused component of the SV image [7], [8]. It would be expected that in case of suppression of blur, sharp adaptation would dominate, and therefore the NPF shift curves would remain mostly at the level of the NPF produced by sharp adaptation. Also, the Maximum Shift Score (Figure 5 B) would have been negative. In case of dominance of the blur component alone, the NPF shift curves will be closer to those of Pure Defocus. NPF and mean Perceptual scores initially increased and then saturated, at 1.2 D for PD and at 0.5 D for SV. Also, our results show that the shift in NPF is highly correlated with the proportion of blur (Figure 2E) and therefore thus does not support the suppression theory. It is possible that the adaptation effects are driven by partial suppression of either of the components or by contrast adaptation.

Changes in the contrast of the natural scenes have been suggested to strongly modulate the state of adaptation, more than differences in the amplitude spectrum frequency of the images [20]. In fact, a proposed function of contrast adaptation or constant gain control is the adjustment of sensitivity to match the prevailing contrast gamut of the image [20]. On the other hand, previous evidence shows that both perceptual judgments of focus and adaptation are controlled by the local blur of the image features, rather than by the global amplitude spectra of the images [17], [18]. This might be the reason why our findings appear better captured by the MSSSIM metric than RMS contrast. We have shown that the aftereffects found in NPF and in the Perceptual Score of image quality correlate significantly with the MSSSIM. In fact, our results (Figure 2F and Figure 5D) show that both for PD and SV images, the adaptation correlates with image quality degradation, indicating similar underlying mechanisms for blur adaptation in both PD and SV images, driven by the effect of blur on local contrast changes in the images.

Our measurements investigate short-term adaptation (60 s) effects to different types of simultaneous blur. However, it is likely that long-term effects are induced by extending the duration of the adaptation period are similar to short term adaptation, as shown in various domains, such as color adaptation [35], adaptation to reduced contrast [36], and adaptation to astigmatic lenses [37]. Whether short-term and long-term adaptations arise from a unique mechanism, or alternatively, different control mechanisms operate at different timescales, as shown for contrast adaptation [38], remains to be seen. However, the observed after-effects following the brief adaptation periods to SV images could persist long-term upon sustained correction, similar to the shift towards isotropy reported by Vinas et al when subjects adapt to their astigmatic correction [12]. Also, adjustments in the gain of the contrast response have been shown following adaptation to reduced contrast by contrast-discrimination measurements and functional Magnetic Resonance Imaging Blood-oxygen-level dependent (fmri BOLD) responses in the visual cortex (V1 and V2) [36]. It is likely that the compensatory perceptual and neural changes produced by a prolonged reduction in retinal image contrast produced in SV images, arise from a response gain mechanism to achieve a contrast gain.

### Visual Performance under Simultaneous Vision

Besides the well-known purposes that adaptation serves in perception (prevention of response saturation, building of a predictive norm-based code, error correction, novelty detection and constancy), it is also interesting to elucidate whether adaptation manifests in improvement of visual performance, usually based on pattern discrimination. A clinical study reported the effect of prior training on visual performance in patients implanted with different types of multifocal intraocular lenses [24]. They reported that visual training to multifocality resulted in significantly better visual performance. Although those effects are sometimes related to perceptual learning [24], i.e. the subject acquiring cues allowing him/her a better response, a recalibration of the internal code for blur as demonstrated by our direct experiments of adaptation (Figure 3A–D and 5A–B), could have played a role in the improvement.

The perceived image quality was worst for a range of near addition around 0.5 D and improved for higher additions. A similar trend in change of decimal visual acuity with SV was noted in a recent study, where decimal visual acuity reached a minimum at a given near addition (2 D addition in that case) and then increased again [25]. While the actual addition range compromising visual quality/perception may vary with the spatial frequency content of the image and the actual task, this observation reinforces that not all near additions in a bifocal correction have equal impact on vision. Very interestingly, we found in this study that after adaptation to simultaneous images with selected near additions, subjects experienced an improvement in perceived image quality of SV images, for all adapting conditions. The adaptation is actually highest for any specific SV correction (defocus component) producing at that specific distance, indicating a full recalibration of the internal code for blur for the correction. Whether this increase in the perceived sharpness after adaptation is also followed by an improvement in visual performance remains to be explored.

### Clinical Implications for Simultaneous Vision Corrections

A presbyopic patient wearing a SV correction and viewing at near will experience much lower blur than that introduced by a single vision lens correcting only for far. In fact, for most subjects and conditions (near additions) images are perceived subjectively less degraded than images degraded by 0.25 D of pure defocus. In addition, we have shown that subjects are able to adapt to the blur produced by a SV correction almost instantly, and it might be possible that this adaptation happens when switching between far and near vision. The close-to-1 slope for SV (in Figure 5C) and the very high statistical significance of the increase indicate that the visual system recalibrates almost fully for each adapting SV image. In a clinical analogue, this will imply that a patient wearing a bifocal correction, fully recalibrates the internal code for blur to that specific correction (regardless of the near addition), thereby achieving maximum perceptual improvement for their conventional working distances. We have also shown that adaptation is selective to each addition and distance. It is also to be noted that different aberrations interact differently with the bifocal correction and this must be taken into account when providing simultaneous vision correction to presbyopic patients. Visual performance under natural viewing conditions could be tested non-invasively using the simultaneous vision system [25] introducing different pupil patterns in the bifocal correction or by actually fitting the bifocal contact lenses.

## Conclusion

The current study addresses visual perception under Simultaneous Vision and provides the first evidence of neural adaptation to bifocal images. We report the following main findings:

1. A shift in the Natural Perceived Focus occurs after adaptation to Pure Defocus and to SV images. This Natural Perceived Focus shift is in concurrence with the magnitude and proportion of defocus.
2. A Simultaneous Vision image with a magnitude of defocus component of 0.5 D is perceived as the most blurred, while images with higher magnitudes of defocus are perceived sharper. The Simultaneous Vision images are always scored higher than PD images (of similar defocus than that of the defocus component in the Simultaneous Vision images). The difference in score of Pure Defocus and Simultaneous Vision is consistent with the differences in local image contrast between both image types.
3. The Natural Perceived Focus and Perceptual Scores shifts correlate significantly with the image quality degradation of the adapting images.
4. The maximum shift in Perceptual Score occurs for the test image with the same amount of defocus as in the adapting image.

In conclusion, perception of bifocal images is partly influenced by the overall blur produced by the correction, and it changes non-monotically with the magnitude of near addition. Even though the image degradation of Simultaneous Vision images was small compared to the Pure Defocus images, subjects are able to adapt to this degradation, as reflected by a shift of the Natural Perceived Focus, and an improvement in the perceived quality following a brief period of adaptation. Therefore, subjects wearing a bifocal correction also experience a spatial calibration of the visual response, following similar mechanisms than those underlying blur adaptation. These adaptation effects are thus important for understanding how vision changes upon a bifocal correction, and may help to define strategies for multifocal lens design and the presbyopic patient management.
